# Erythrocytes and Erythropoietin

**DOI:** 10.1155/2011/972536

**Published:** 2012-02-28

**Authors:** Michael Föller, Lars Kaestner, Elisabetta Straface, Johannes Vogel

**Affiliations:** ^1^Department of Physiology, University of Tübingen, Gmelinstra*β*e 5, 72076 Tübingen, Germany; ^2^Institute for Molecular Cell Biology, School of Medicine, Saarland University, 66421 Homburg/Saar, Germany; ^3^Section of Cell Degeneration and Gender Medicine, Department of Therapeutic Research and Medicines Evaluation, Istituto Superiore di Sanità, Viale Regina Elena 299, 00161 Rome, Italy; ^4^Institute of Veterinary Physiology, Vetsuisse Faculty and Zürich Center for Integrative Human Physiology, University of Zürich, 8057 Zürich, Switzerland

This special issue focuses on erythrocytes and erythropoietin including original research and review articles on the cellular physiology of erythrocytes and of erythrocyte-associated disorders. Moreover, the special issue includes papers on the action of erythropoietin and hypoxia-inducible factors on a cellular level. In the following, we present the original papers published in this special edition.

D. Barneaud-Rocca et al. deal with one of the most abundant proteins in red blood cells, the band 3 protein (Anion Exchanger 1). The paper reviews recent work hypothesizing the contribution of band 3 point mutations to a sodium and potassium leak. It is compared to alternative explanations suggesting that point mutations in band 3 regulate the cation leak through other transporters. The topic is driven by the fact that mutations in the band 3 protein have been associated with hereditary stomatocytosis. The molecular mechanisms discussed in the paper link the stomatocytosis and the sodium potassium leak of the mutated band 3 protein.

W. Nunomura et al. review the function of unstructured N-terminal domain of protein 4.1R and 4.1 G and characterize the binding profiles of proteins 4.1R80, 4.1R135 and protein 4.1G in erythropoiesis. The regulation of the binding profiles of these proteins by the presence or absence of the N-terminal 209 amino acid sequence (headpiece region (HP)) and unstructured domain of the protein as well as of 4.1R135 (which contains the HP) by both Ca^2+^ and Ca^2+^/CaM is discussed. Knowing the different regulation and expression of the 4.1 protein isoforms will foster our understanding of erythropoiesis.

F. Fares et al. fused one Carboxyl-Terminal Peptide (CTP) of the human chorionic gonadotropin beta subunit to the N-terminal end and two CTPs to the C-terminal end of erythropoietin (Epo). This artificial erythropoiesis-stimulating agent had increased *in vivo* activity as well as half-life compared with recombinant human Epo and the hyperglycosylated Epo analogue darbepoetin alfa (Aranesp). As erythropoiesis-stimulating agents often need to be injected repeatedly over long time periods, for example, for anemia treatment in kidney diseases or cancer, more effective and longer lasting Epo derivates improve the patients' quality of life.

E. Straface et al. present the results of a pilot study investigating new peripheral sex-associated markers in patients with metabolic syndrome and subclinical atherosclerosis. The metabolic syndrome, which is characteristic of hypertension, obesity, insulin resistance, hypertriglyceridemia, and hypercholesterolemia, is a major risk factor for cardiovascular mortality in the developed world. In their study, E. Straface et al. analyzed glycophorin A, CD47, and phosphatidylserine exposition at the cell surface as hallmarks of erythrocyte damage. They report significant gender differences of those parameters in patients with metabolic syndrome.

L. J. Norton et al. review the recent advance in cellular reprogramming with the particular emphasis on its potentially beneficial use for the future treatment of hemoglobinopathies. Typical and frequent hemoglobinopathies are genetic disorders such as thalassaemia and sickle cell disease. To date, blood transfusion is the principal therapy of those diseases. In their review article, L. J. Norton et al. outline the recent development in stem cell research such as classic cellular reprogramming and transdifferentiation and discuss their potential application for the treatment of anemia following hemoglobinopathies.

We hope that this special issue will alert researchers to some recent development in the field of erythrocytes and erythropoietin, particularly the correlation between protein alterations and clinical symptoms, and that a better understanding of this correlation can direct our efforts to the discovery of new therapeutic strategies for the treatment of anemia and metabolic disorders.

*Michael Föller* *Michael Föller* *Lars Kaestner* *Lars Kaestner* *Elisabetta Straface* *Elisabetta Straface* *Johannes Vogel* *Johannes Vogel*

